# Pathological Complete Response and Successful Conversion Hepatectomy in Initially Unresectable Giant Hepatocellular Carcinoma Following Triple Therapy With HAIC, Bevacizumab, and Tislelizumab: A Case Report

**DOI:** 10.1002/ccr3.73835

**Published:** 2026-11-04

**Authors:** Qing Ye, Jiamin Wang, Junming He, Jianxin Peng, Huilan Zeng, Jiaqiang Mo

**Affiliations:** ^1^ Department of Hepatobiliary Surgery Guangdong Provincial Hospital of Chinese Medicine, The Second Affiliated Hospital of Guangzhou University of Chinese Medicine Guangzhou China; ^2^ The Second Clinical College of Guangzhou University of Chinese Medicine Guangzhou China

**Keywords:** conversion therapy, future liver remnant, giant hepatocellular carcinoma, hepatic arterial infusion chemotherapy, pathological complete response

## Abstract

Severe future liver remnant (FLR) deficiency frequently renders giant hepatocellular carcinoma (HCC) unresectable. We report a 48‐year‐old man with an unresectable 23.5‐cm giant HCC (Couinaud segments 4/5/8; baseline serum alpha‐fetoprotein (AFP), 1036.65 μg/L). Initially requiring an extended right trisectionectomy with an FLR/standard liver volume (SLV) ratio of only 36.58% (450 mL). Following three cycles of mFOLFOX‐HAIC, bevacizumab, and tislelizumab, restaging computed tomography (CT) showed tumor regression to 9.0 cm in maximum diameter (a 61.7% reduction), corresponding to a partial response (PR) according to the Response Evaluation Criteria in Solid Tumors, version 1.1 (RECIST 1.1), and a complete response (CR) according to the modified RECIST (mRECIST), an increase in FLR volume to 733 mL (FLR/SLV: 59.58%), and an AFP decline to 115.00 μg/L. Crucially, tumor clearance from segment 4 and separation from the right portal pedicle enabled surgical downstaging to a parenchymal‐sparing anatomical right anterior sectionectomy (S5/8). R0 resection was achieved, with histopathology confirming a pathological complete response (pCR; 100% coagulative necrosis). Postoperatively, serum AFP normalized to 5.60 μg/L, and the patient remains recurrence‐free at 22 months. This case illustrates that tumor regression during triple therapy may be accompanied by increased FLR volume and improved anatomical separation from the right portal pedicle, potentially enabling a more limited resection without portal vein embolization.

## Key Clinical Message

In initially unresectable giant HCC with an inadequate FLR, triple conversion therapy with mFOLFOX‐HAIC, bevacizumab, and tislelizumab was followed by marked tumor regression, an increase in the FLR/SLV ratio from 36.58% to 59.58%, and pathological complete response, enabling R0 resection.

## 1 Introduction

Hepatocellular carcinoma (HCC) is a common primary liver malignancy. While surgical resection remains the primary curative modality for early‐stage HCC, most patients present beyond the window for upfront resection at initial diagnosis [1, 2]. In patients with giant HCC (> 10 cm) without extrahepatic metastasis or macrovascular invasion, an inadequate future liver remnant (FLR) volume is the main cause of technical unresectability [3], posing a high risk of post‐hepatectomy liver failure (PHLF) [4]. Although traditional portal vein occlusion strategies—such as portal vein embolization (PVE) and associating liver partition and portal vein ligation for staged hepatectomy (ALPPS)—can induce FLR hypertrophy, their clinical utility is limited by tumor progression during the waiting period and substantial surgical invasiveness [5, 6]. Recently, multimodal conversion regimens combining hepatic arterial infusion chemotherapy (HAIC) with immunotherapy and anti‐angiogenic agents have shown promising conversion efficacy by inducing rapid tumor shrinkage and secondary compensatory FLR expansion [7, 8].

Atezolizumab plus bevacizumab has been shown to improve overall survival (OS) compared with sorafenib in unresectable HCC [9]. Additionally, a retrospective study demonstrated that adding mFOLFOX‐HAIC to tislelizumab plus lenvatinib significantly increased the objective response rate (ORR) from 46.7% to 78.8% in large HCC with portal vein tumor thrombosis (PVTT) [10]. However, whether the triple regimen of mFOLFOX‐HAIC, bevacizumab, and tislelizumab can induce deep tumor regression and achieve a pathological complete response (pCR) in giant HCC unresectable solely due to FLR insufficiency has not been previously reported.

Tumor response in this case was evaluated according to Response Evaluation Criteria in Solid Tumors, version 1.1 (RECIST 1.1) [11] and modified RECIST (mRECIST) [12]. Here, we report a patient with a 23.5‐cm giant HCC who achieved a pCR and underwent successful R0 resection following conversion therapy with this triple regimen, remaining recurrence‐free at 22 months postoperatively.

## 2 Case Presentation

### 2.1 Baseline Characteristics and Initial Unresectability Assessment

A 48‐year‐old male with a 20‐year history of chronic hepatitis B on entecavir presented on November 10, 2023, following the incidental detection of a giant hepatic mass. Contrast‐enhanced computed tomography (CT) revealed a giant nodular hepatocellular carcinoma (HCC) predominantly involving Couinaud segments 4, 5, and 8 (Figure 1A,B,D) (23.5 × 12.3 × 19.5 cm), exerting marked mass effect on the adjacent liver parenchyma and portal hilum (Figure 1C). Baseline serum alpha‐fetoprotein (AFP) was markedly elevated at 1036.65 μg/L. The patient had preserved liver function (Child‐Pugh class A), an Eastern Cooperative Oncology Group performance status (ECOG PS) of 1, and China Liver Cancer (CNLC) stage Ib and Barcelona Clinic Liver Cancer (BCLC) stage A disease, without macrovascular invasion or extrahepatic spread.

**FIGURE 1:**
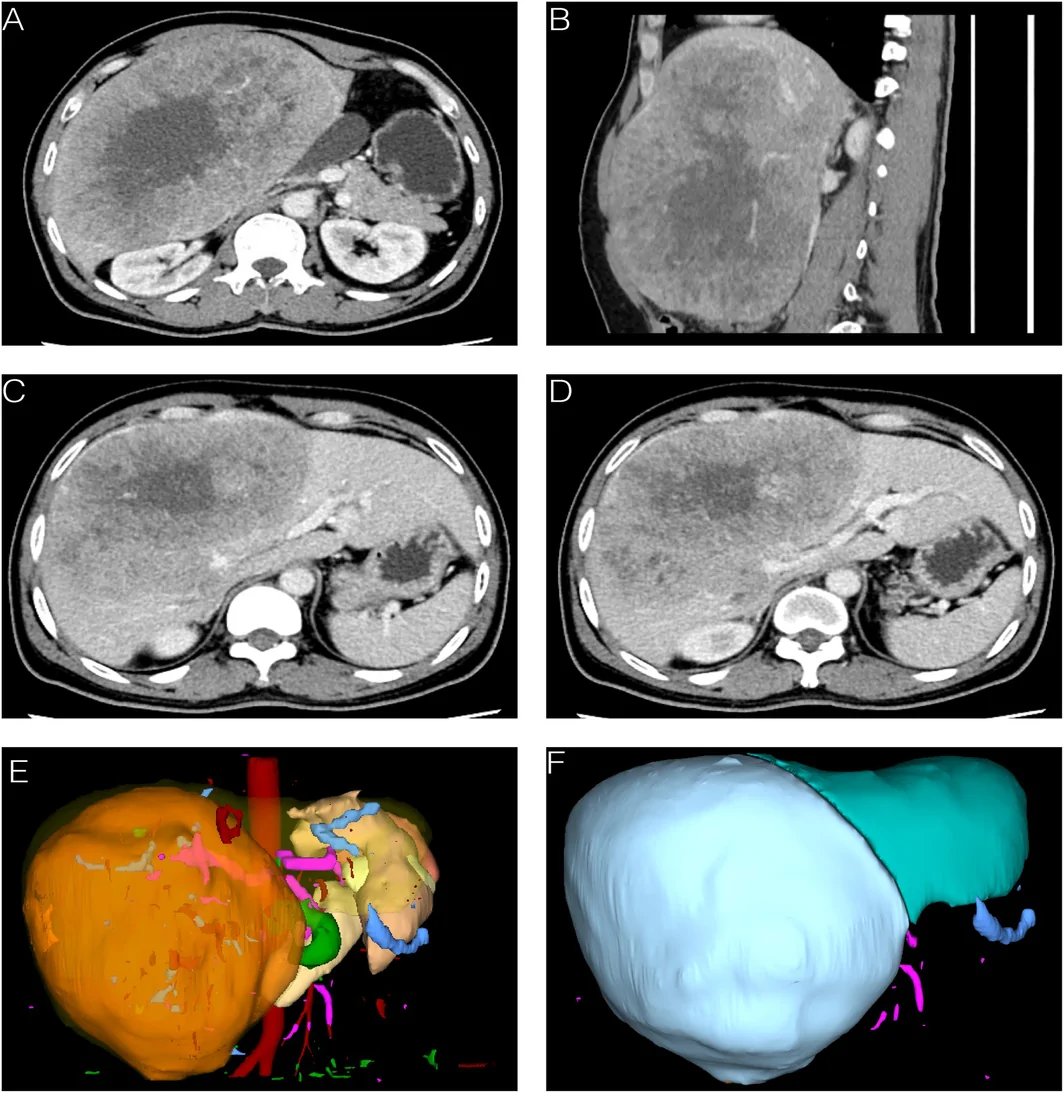
Pretreatment contrast‐enhanced CT and surgical planning for giant HCC. (A, B) A massive tumor involving segments 4, 5, and 8 (S4/5/8), measuring 23.5 cm in maximum transverse diameter (A) and 19.5 cm in longitudinal diameter (B). (C) Tumor compression on the main right portal vein trunk. (D) Segment 4 (S4) occupied by the tumor. (E) Three‐dimensional (3D) reconstruction demonstrating the hepatic vasculature and the tumor's relationship with the portal vein. (F) Three‐dimensional volumetry for the planned right trisectionectomy showing a future liver remnant (FLR) of 450 mL, corresponding to 36.58% of the standard liver volume (SLV).

However, three‐dimensional (3D) CT volumetry demonstrated that upfront curative resection was unfeasible due to critical FLR insufficiency (body surface area: 1.73 m^2^; standard liver volume [SLV]: 1230 mL; total liver volume: 1846 mL). A right trisectionectomy would leave an FLR consisting only of the left lateral section, measuring 450 mL (an FLR/SLV ratio of 36.58%). Given the planned extended right trisectionectomy, this FLR volume was considered insufficient to meet the institutional safety threshold for this extensive hepatectomy, with a high risk of post‐hepatectomy liver failure. Given the prohibitive risk of PHLF and the inseparable tumor–portal vein adhesion on imaging, the tumor was deemed initially technically unresectable, and a right trisectionectomy would have been the only potential R0 resection strategy (Figure 1E,F).

### 2.2 Multimodal Conversion Therapy and Treatment Response

Following multidisciplinary team (MDT) consultation, the patient initiated triple conversion therapy on November 16, 2023, receiving three 3‐week cycles of HAIC using a modified FOLFOX regimen (mFOLFOX; oxaliplatin 85 mg/m^2^, leucovorin 400 mg/m^2^, fluorouracil bolus 400 mg/m^2^ on Day 1, and fluorouracil infusion 2400 mg/m^2^ for 48 h, once every 3 weeks), combined with bevacizumab (7.5 mg/kg) and tislelizumab (200 mg) every 3 weeks. Bevacizumab was dose‐reduced to mitigate bleeding risks from mild baseline esophagogastric varices. Treatment concluded on January 1, 2024, without grade ≥ 3 adverse events.

Restaging CT 2 weeks post‐treatment (January 15, 2024) confirmed major tumor downstaging to 9.0 × 7.1 × 5.8 cm (a 61.7% decrease; RECIST 1.1 PR and mRECIST CR) with extensive necrosis and loss of hypervascularity (Figure 2A,B). Concurrently, 3D volumetry documented FLR expansion from 450 mL to 733 mL (FLR/SLV 59.58%) (Figure 2E,F). Importantly, the mass had separated from the right posterior Glissonian pedicle with complete clearance of Segment 4 (Figure 2C,D), making parenchymal‐sparing resection anatomically feasible.

**FIGURE 2:**
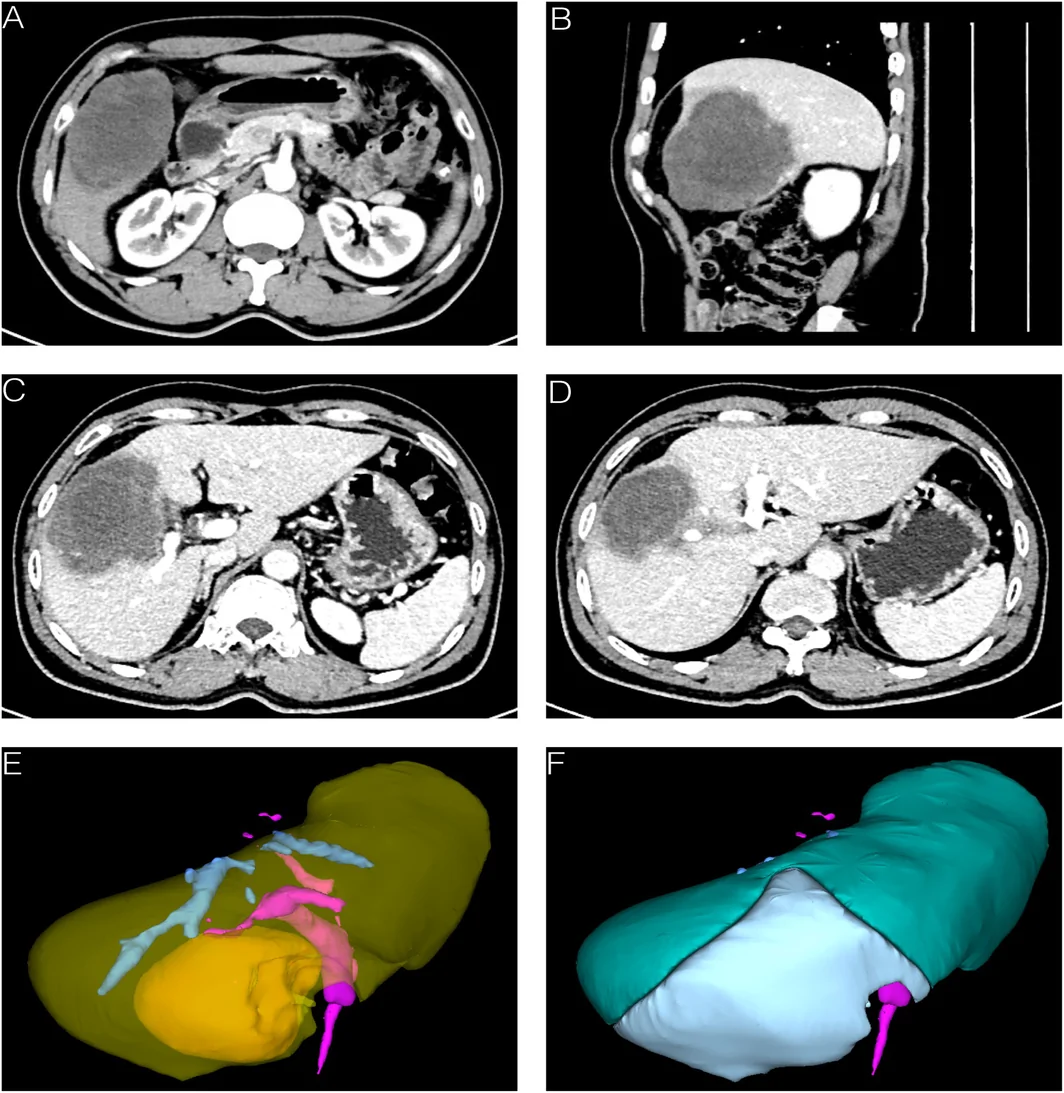
Contrast‐enhanced CT following three cycles of conversion therapy. (A, B) Significant tumor regression, with the residual mass localized to segments 5 and 8 (S5/8), measuring 9.0 cm (transverse) (A) and 7.1 cm (longitudinal) (B). (C) Post‐treatment demarcation showing a well‐defined boundary between the tumor and the adjacent right posterior Glissonian pedicle. (D) Segment 4 (S4) free of tumor involvement after shrinkage. (E) 3D reconstruction showing the preserved hepatic vasculature after tumor regression. (F) 3D volumetry for a planned right anterior sectionectomy (preserving the right posterior section and left hemiliver), yielding an FLR (right posterior section + left hemiliver) of 733 mL (FLR/SLV = 59.58%).

### 2.3 Surgical Intervention

On February 2, 2024 (32 days post‐conversion), the patient underwent elective open anatomical right anterior sectionectomy (S5/8 bisegmentectomy) under general anesthesia. Based on the restaging findings of tumor detachment from the right portal pedicle and clearance of Segment 4, the originally planned right trisectionectomy was revised to this parenchymal‐sparing procedure. Intraoperative exploration revealed an encapsulated, firm mass (9 × 8 × 8 cm) with dense adhesions to the anterior abdominal wall and omentum, which were safely lysed. Guided by intraoperative ultrasonography to define the tumor margins and the middle hepatic vein, parenchymal transection was performed along the plane between the middle and right hepatic veins under an intermittent Pringle maneuver (two cycles, 15 and 14 min; total ischemia time: 29 min). An en bloc R0 resection was achieved with negative margins. Total operative time was 3 h and 35 min, with no blood transfusions required.

### 2.4 Histopathological Examination and Pathological Complete Response

Gross examination of the resected specimen revealed a 10.5 × 9.8 × 7.5 cm grayish‐yellow nodular tumor bed. Serial whole‐mount sectioning at 5‐mm intervals, evaluated independently by two senior pathologists, demonstrated 100% tumor necrosis with surrounding fibrosis, focal hemorrhage, and chronic inflammatory infiltration. No viable neoplastic cells or margin involvement were identified, confirming a pCR (Figure 3A,B).

**FIGURE 3:**
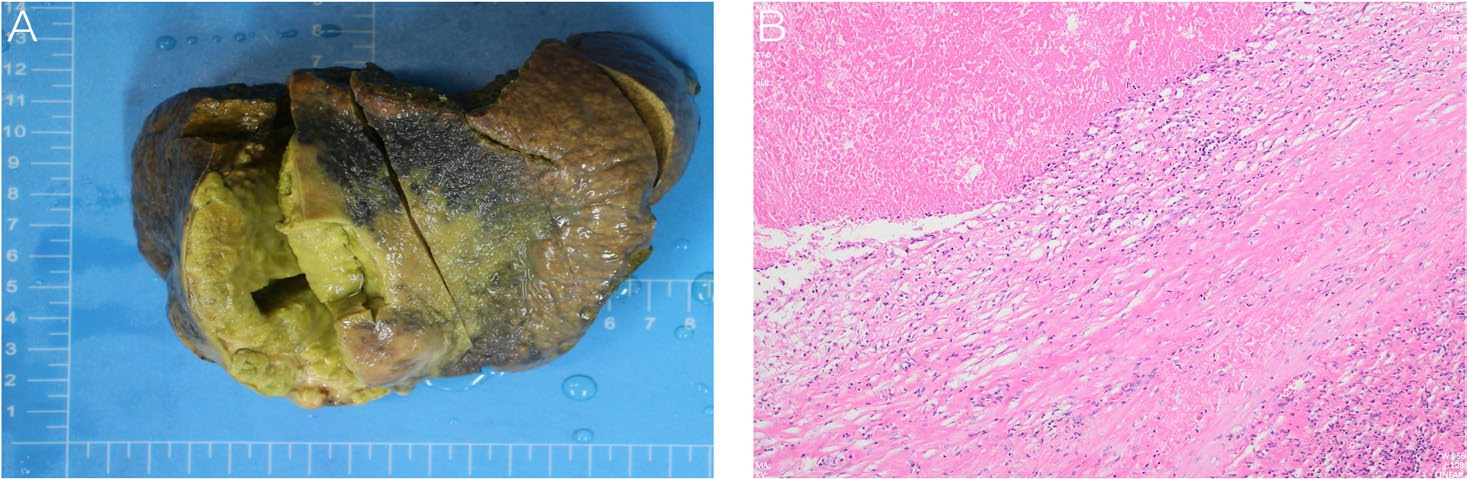
Macroscopic and microscopic features of the resected tumor specimen. (A) Gross specimen of the resected liver showing an encapsulated nodular tumor bed (10.5 × 9.8 × 7.5 cm) with extensive central coagulative necrosis. (B) Microscopic examination (hematoxylin and eosin staining) demonstrating complete tumor necrosis accompanied by dense fibrous proliferation and chronic inflammatory infiltrate, with no viable tumor cells.

### 2.5 Outcome and Follow‐Up

The patient had an uneventful recovery without surgical complications or hepatic dysfunction and was discharged on postoperative Day 14. Follow‐up CT at 1 month showed an intact resection margin without local recurrence or distant metastasis, with serum AFP normalizing to 5.60 μg/L. At 22 months postoperatively (December 2025), the patient remains disease‐free with an ECOG PS of 0 and normal serum tumor markers (Figure 4A,B).

**FIGURE 4:**
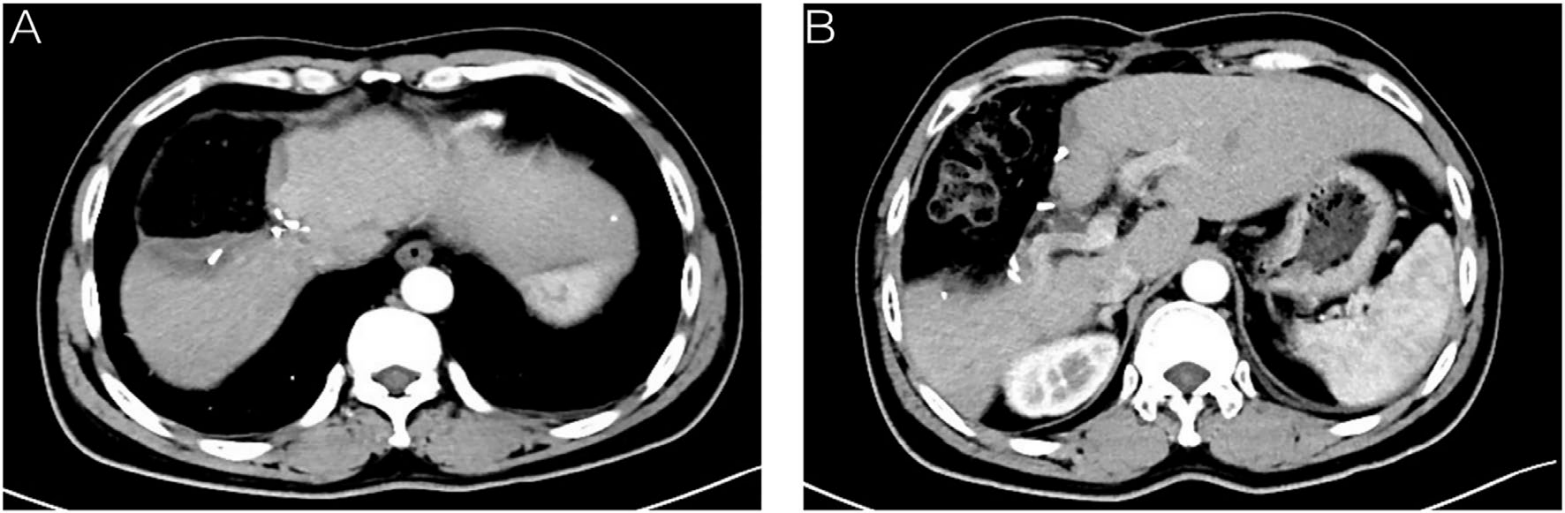
Follow‐up CT under active surveillance at 22 months postoperatively showing no tumor recurrence. (A, B) Arterial phase demonstrates patent hepatic arterial and venous blood flow, with no new enhancing nodules in the hepatic parenchyma.

## 3 Discussion and Conclusion

In patients with giant or multifocal HCC, an inadequate FLR and the heightened risk of PHLF substantially limit the applicability of curative‐intent surgical resection [13].

Conventional transarterial chemoembolization (cTACE) has shown limited efficacy in large or giant tumors; a retrospective cohort of 149 patients with large HCC (≥ 5 cm) reported an ORR of 14.0%–34.0% following cTACE [14]. In contrast, a retrospective study showed that a triple regimen combining mFOLFOX‐HAIC, bevacizumab, and a programmed cell death protein 1 (PD‐1) inhibitor enabled successful surgical conversion in 83.3% of initially unresectable HCC cases, with 33.3% achieving a pCR [15]. Our therapeutic strategy harnesses local HAIC‐induced tumor necrosis alongside anti‐angiogenic and immune activation to drive profound tumor regression. This case provides an additional clinical observation of a pathological complete response following conversion therapy with mFOLFOX‐HAIC, bevacizumab, and tislelizumab in giant HCC initially considered unresectable because of insufficient FLR volume.

Beyond dimensional reduction, the therapeutic response in this case enabled improved anatomical separation—the regressed tumor separated from the right portal pedicle, and Segment 4 was completely cleared of involvement. This anatomical reconfiguration allowed conversion from an extended right trisectionectomy (which would have left a marginal FLR) to a limited S5/8 segmentectomy, ultimately preserving the right posterior sector and significantly augmenting functional FLR. This surgical downstaging—from a technically challenging extended resection to a more limited parenchymal‐sparing procedure—underscores that a profound pathological response can translate into tangible surgical benefits beyond radiological downsizing alone.

In the present case, post‐treatment CT following the triple regimen showed a PR by RECIST 1.1 but a CR by mRECIST, while pathology confirmed a pCR. This discrepancy highlights RECIST 1.1's tendency to underestimate actual tumor necrosis, echoing previous reports where 10 of 24 RECIST‐PR patients (41.7%) had no viable tumor on histology [16]. Treatment‐induced inflammation and fibrosis frequently cause persistent enhancement, creating false‐positive impressions of viable tumor. Therefore, surgical eligibility should not be determined solely by RECIST 1.1 radiologic response; comprehensive clinical and anatomical assessment is mandatory.

Several limitations of this report merit acknowledgment. First, as a single‐case observation, the efficacy and safety of this triple regimen require validation in prospective, large‐scale cohorts. Second, given that this patient had hepatitis B virus (HBV)‐related HCC, whether comparable benefits translate to non‐viral etiologies remains to be determined. Finally, extended postoperative follow‐up is essential to comprehensively evaluate long‐term recurrence outcomes.

In conclusion, triple conversion therapy with mFOLFOX‐HAIC, bevacizumab, and tislelizumab demonstrated substantial downstaging capability and the potential to achieve pCR in giant HCC unresectable solely due to FLR insufficiency, representing a promising conversion strategy that warrants further clinical evaluation.

## Author Contributions

**Qing Ye:** writing – original draft. **Jiamin Wang:** writing – original draft. **Junming He:** writing – original draft. **Jianxin Peng:** writing – original draft. **Huilan Zeng:** writing – original draft. **Jiaqiang Mo:** writing – original draft.

## Funding

This study was supported by the 2025 Traditional Chinese Medicine Research Project of the Guangdong Provincial Administration of Traditional Chinese Medicine, (No. 20251182), the Hospital‐Level Special Research Project of Guangdong Provincial Hospital of Chinese Medicine, (No. YN2024MS069), the National Natural Science Foundation Cultivation Project of Guangdong Provincial Hospital of Chinese Medicine, (No. YN2025GZR43), the Key Laboratory of Traditional Chinese Medicine Syndrome Clinical Research of Guangdong Province, (No. KF2023ZH01), the Guangzhou Municipal Science and Technology Bureau Municipal‐Hospital Joint Funding Project, (No. 2025A03J2347).

## Ethics Statement

This study was approved by the Clinical Research Ethics Committee of Guangdong Provincial Hospital of Chinese Medicine (No. YE2024‐377‐01), and written informed consent was obtained from the patient for publication of this case report and any accompanying images.

## Conflicts of Interest

The authors declare no conflicts of interest.

## Data Availability

The authors have nothing to report.
